# Desolvation Inability of Solid Hydrates, an Alternative Expression for the Gibbs Free Energy of Solvation, and the Myth of Freeze-Drying

**DOI:** 10.3390/ma17112508

**Published:** 2024-05-23

**Authors:** Costas Tsioptsias

**Affiliations:** Department of Chemical Engineering, University of Western Macedonia, 50132 Kozani, Greece; aff00285@uowm.gr or ktsiopts@gmail.com

**Keywords:** solvation, hydrate, Gibbs free energy, gallic acid, magnesium chloride

## Abstract

The term “desolvation inability” is proposed in order to describe the alteration of the original chemical structure of a solute (“decomposition”) prior to the solvent’s full removal upon the heating of the solvate. This behavior has been sporadically reported; however, it is much more frequent, and it is the basis of various, seemingly unrelated, effects/processes, e.g., the vinegar syndrome of cellulose acetate cinematographic films, in thermal energy storage. An explanation and a criterion/index for the prediction of this behavior are provided based on the comparison of the Gibbs free energies of decomposition and desolvation. A new approach for the expression of the Gibbs free energy of desolvation is proposed by reversing the roles of the solute and solvent and by regarding water as the solute rather than as the solvent, while the solute is treated as a solid solvent. This approach results in lower solvation/desolvation Gibbs free energy values. Based on the above, the experimentally observed thermal behavior of three inorganic hydrates is predicted and explained. Theoretically and experimentally, it is supported that decomposition is possible at sub-zero (°C) temperatures and the regarded simultaneous drying and protection of heat-sensitive substances by freeze-drying, at least in some cases, e.g., for the case of gallic acid, is an unverified myth.

## 1. Introduction

Solvation/desolvation and the behavior of solvates are important phenomena which are involved in various processes and applications, such as protein folding, molecular recognition and ligand binding [1], active ingredient production (e.g., for foods or pharmaceuticals), geoscience [2] and hydrophobic hydration/solvation [3], storage of thermal energy [4,5], etc. Thus, it is not surprising that an increased theoretical and modeling interest for the effect of solvation exists in the literature [1,6]. Hydration is a special case of solvation in which water is the solvent. The dehydration process is quite different depending on the environment in which it proceeds and the Gibbs free energy of desolvation does not necessarily equal to the one of solvation, e.g., the release of hydrated water in the bulk solution may be accompanied by zero Gibbs free energy of desolvation due to entropy–enthalpy compensation [7], though this is not a universal effect [6].

Galwey, decades ago, pointed out that during the interpretation of experimental results for the dehydration of both inorganic and organic hydrates, it should be taken into account that various reactions may occur during heating, including a hydrolysis reaction, as it occurs in MgCl_2_ dihydrate [8]. More precisely, only half of the water is removed while the other water molecules react with MgCl_2_ to give MgO and HCl [8]. MgCl_2_ hexahydrate releases water gradually and can be transformed to MgCl_2_ dihydrate. MgOHCl is formed as an intermediate product of MgCl_2_ hydrolysis and all these reactions have been used for thermal energy storage [5].

In other words, according to Galwey [8], it should not be taken for granted that, in organic and inorganic hydrates, upon heating, water will be removed from any solute. However, in various cases, water removal is taken for granted. Some of these cases concern organic substances, e.g., the broad endothermic peak in the differential scanning calorimetry (DSC) curve of cellulose esters around 100 °C, which for decades has been typically erroneously attributed solely to water evaporation, but recently it was shown to arise mainly from the esterification reaction between free acetic acid and the OH groups of cellulose esters [9]. This actually is the reverse process which is responsible for the vinegar syndrome of cinematographic films. More precisely, once cellulose acetate absorbs water, some hydrolysis occurs leading to the formation of free acetic acid. If heating is applied in order to remove acetic acid, esterification occurs and leads to the production of new water. Upon cooling, new acetic acid will be formed, and a vicious cycle is generated. It is rather impossible to get rid of the impurities from cellulose acetate by heating, e.g., it has been reported that even after 5 days of drying at 125 °C, the broad endothermic peak around 100 °C was still present in the DSC curve of cellulose acetate [10]. Seemingly, there is little relation between this effect and the decomposition of MgCl_2_ hydrate which is used for thermochemical energy storage applications. Similarly, gallic acid (3,4,5-trihydroxy benzoic acid) exhibits an endothermic peak close to but below 100 °C. This, for years, has been interpreted either as water removal or as a solid–solid transformation. However, the thermal behavior of gallic acid was recently clarified and it was shown that it decomposes around 90 °C without full water removal [11]. The absence of knowledge of the desolvation inability is most likely the hidden cause of these long-standing misinterpretations.

In other words, the decomposition of the solute prior to the full removal of the solvent has been observed both for organic and inorganic substances, but in various cases it is taken for granted that full water removal is possible and erroneous interpretations have been provided. An additional source of confusion might be the use of the term “decomposition of hydrate” for both cases: (a) actual decomposition of the solute and (b) the “decomposition” of the hydrate into water and the anhydrous form. For example, in the *Handbook of Chemistry and Physics* [12], in the table with the melting point data, one can find numerous cases of hydrates where, in the melting point, it is clarified that decomposition occurs. However, it is not specified if the decomposition is an actual one or refers to the formation of the solute in the anhydrous form. The introduction of the term “desolvation/dehydration inability” perhaps might assist in giving the proper attention to this kind of thermal behavior, which seems to be broad and expand beyond some inorganic salts. The scope of this work is to provide a thermodynamic basis for this kind of thermal behavior and to propose a criterion that can be used for predicting dehydration ability/inability. In order to accomplish this, an alternative approach for the expression of Gibbs free energy of solvation/desolvation is proposed by considering water as the solute (and not as a solvent). The existence of the desolvation inability in the case of freeze-drying is also examined.

## 2. Materials and Methods

Gallic acid (G7384, purity > 97.5%) and magnesium chloride hexahydrate (MgCl_2_·6H_2_O, purity > 99%) were purchased from Sigma (St. Louis, MO, USA) while potassium bromide (KBr) of purity > 99.5% was purchased from Chem-Lab (Zedelgem, Belgium). Fourier transform infrared (FTIR) spectroscopic and X-ray diffraction (XRD) measurements were conducted, respectively, in a Thermo (Waltham, MA, USA) FTIR spectrometer and a Bruker (Billerica, MA, USA) D8 Advance diffractometer with a Siemens Cu X-ray tube emitting radiation of 1.54 Å. A Christ freeze-drier (model Gamma 1–20) was also used. KBr was dried at 140 °C for 1 h prior to use, while all other chemicals were used as received. Gallic acid and MgCl_2_·6H_2_O were freeze-dried for 21 h. The raw and the freeze-dried gallic acid samples were studied by FTIR with the KBr method (64 scans, resolution of 2 cm^−1^). The raw and the freeze-dried MgCl_2_·6H_2_O samples were examined by XRD at room conditions. In the FTIR spectra baseline, correction was applied. In both FTIR spectra and XRD graphs, normalization in scale 0–1 was performed.

## 3. Theoretical Analysis

In this section, initially, an explanation and criterion will be provided for the desolvation inability. Some more specific aspects regarding the Gibbs free energy of solvation/desolvation then will be discussed. Finally, the calculation details for applying the criterion for three case studies will be presented. These three cases studies concern hydrates of NaCl, CaCl_2,_ and MgCl_2_. Before proceeding, it should be stressed that there is no reason to assume that the desolvation inability is limited only to hydrates. The proposed criterion should be valid for all solvates. Here, we simply focus on hydrates due to their increased involvement in various applications. Also, the theoretical analysis is limited to inorganic salts, only for a practical reason, that is, there are various available data, e.g., enthalpies of formation, of solvation, etc. For organic substances like gallic acid, no such data could be found. The same holds for some inorganic substances which are intermediate products, e.g., MgOHCl or hydrate forms of MgCl_2_.

Before proceeding the following clarification should be made. The criterion is based on the comparison of the Gibbs free energy of decomposition and desolvation. It is stressed that the term “decomposition” is used in a very broad sense in order to describe any chemical reaction that results in the alteration of the original chemical structure of the solute. For example, let us suppose that like gallic acid, an amino-acid hydrate, upon heating, instead of dehydration, undergoes a condensation reaction which leads to the formation of a dipeptide. This dimerization reaction, obviously, from the point of view of biology, is not a decomposition reaction; on the contrary, it is the primary reaction for protein synthesis. However, from the point of view of thermodynamics, this reaction “competes” with dehydration and leads to an alteration of the original structure of the solute upon heating. Here we focus on the alteration of the original chemical structure of the solute, and other issues, e.g., the exact nature of the chemical reaction is considered a secondary issue. Similarly, the extent of the chemical reaction that occurs is also a secondary issue. Thus, the term “decomposition” is abusively used in order to describe any reaction that results in the alteration of the chemical structure of the solute, regardless of the nature of the reaction and its extent. Thus, reactions such as hydrolysis, dimerization, cyclization, etc. are also included in the term “decomposition”.

### 3.1. Criterion for the Desolvation Inability

A concept similar to the one which was reported recently for the prediction of the melting inability in molecular solids [13] will be adopted here to describe the behavior of desolvation inability. More precisely, based on the second law of thermodynamics, it is taken for granted that if a solvate exhibits solvation inability it is because the Gibbs free energy of decomposition of the solute is lower than the Gibbs free energy of desolvation. Thus, such solutes prefer to be decomposed rather than to be separated from the solvent. This can be used to develop a criterion for predicting the desolvation behavior (ability/inability) of a certain solvate/hydrate as follows:

(1) $$\begin{array}{c} DI=\Delta G_{desolvation}-\Delta G_{decomposition}\\ if\;DI<0\;\Rightarrow desolvation\;ability\\ if\;DI\geq 0\;\Rightarrow desolvation\;inability \end{array}$$

where

DI: desolvation inability index;

$\Delta G_{desolvation}$: Gibbs free energy of desolvation;

$\Delta G_{decomposition}$: Gibbs free energy of decomposition (refers to the pathway with the lowest Gibbs free energy in case there are multiple pathways for decomposition).

It would be ideal to also know the Gibbs free energies of activation for desolvation and decomposition, so as to be able to compare the respective rates in which the $\Delta G_{desolvation}$ and $\Delta G_{decomposition}$ are practically the same. However, here we will examine the desolvation inability focusing on the thermodynamics and not the kinetics of solvation and decomposition.

It should be stressed, again, that the term decomposition is used here to describe any chemical reaction that alters the chemical structure of the solute. Provided that the enthalpies of formation and molar entropies of the reactants and the products are known, then the $\Delta G_{decomposition}$ can be calculated. For the calculation of the other term ($\Delta G_{desolvation}$), one might be tempted to use the negative value of Gibbs free energy of solvation for aqueous solutions, that is as follows:

(2) $$\Delta G_{desolvation}=-\Delta G_{solvation,liquid\;solution}\;\;\left(not\;applicable\;in\;Equation\;\left(1\right)\right)\;$$

where

$\Delta G_{solvation,liquid\;solution}$: Gibbs free energy of solvation of a solute during the formation of liquid solution.

We believe that this is not correct and for this reason, the next section is dedicated to the proposition of an alternative approach for the estimation of $\Delta G_{desolvation}$, without, however, affecting the definition of solvation.

### 3.2. How Many Gibbs Free Energies of Solvation Exist for the Same System?

The solvation of a solute by an excess number of solvent molecules in a liquid solution and the solvation of the same solute in the solid state by an extremely limited number of the same solvent molecules, e.g., in mono-hydrates, penta-hydrates, etc., are two quite different states and demand a quite different handling.

According to the Gold Book of IUPAC [14], the Gibbs free energy (and the enthalpy and entropy as well) of solvation is defined as the free energy change for bringing a molecule of the solute from a vacuum or the gas phase in the solvent. For organic (volatile) molecules, this can be calculated from the free energies of condensation (vaporization) and solution [12]. For inorganic electrolytes, the Gibbs free energy of solvation is related to the lattice energy of the solute and the free energy of solution. In other words, the energy of solvation is the sum of the energy that is needed to be absorbed, in order to bring the solute from the gas to the liquid or solid state (minus the energy of vaporization or lattice energy) and the energy which is absorbed or typically liberated in order to dissolve the solute in the solvent. This is totally meaningful if one wants to study aqueous (or other liquid) solutions. For example, in order for hydration to occur in aqueous solution, then the lattice energy must be provided and the crystalline lattice of the solid must collapse. However, for the case of desolvation of a solid inorganic hydrate (and not the solvation in aqueous solution), maybe the hydrated and dehydrated solute crystallize in different types, but the lattice of the solute is already formed to a great extent since it is in the solid state. Thus, in order to dehydrate a solid hydrate, the solute–solvent interactions must be broken (related to the free energy of solution) but there should not be any involvement of the solute’s total lattice energy. Similarly, for an organic solid hydrate, e.g., gallic acid hydrate, in order to dehydrate it, the interactions between gallic acid and water should be broken (related to the free energy of the solution), but the energy of gallic acid vaporization should not be involved in the dehydration process as it occurs during the solvation of gallic acid in liquid aqueous solution. In both of these cases, it seems that the lattice energy or the energy of vaporization of the solute must be replaced by the energy of vaporization of water (solvent). This is in full agreement with the definition of solvation, as long as we make some reversal in the definitions of our system. In solid hydrates, the substance with the largest portion (in terms of mass) is the solute, while the solvent (water) is the compound with the lowest portion. In aqueous solutions, the electrolyte’s ions or the organic substances’ molecules are surrounded by water molecules, while in solid hydrates the water molecules are surrounded by the ions or large organic molecules. In any case, we have the right to consider/define the electrolyte or the organic molecule as the solvent and consider water as the solute. By this reversal in the solute–solvent definitions and by using the established definition of Gibbs free energy of solvation, it follows that for a solid hydrate, the Gibbs free energy of solvation is the sum of the free energy that is involved when the solute (water) from gas converts to liquid (that is, the negative value of the free energy of vaporization) and the free energy of the solution, that is as follows:

(3) $$\Delta G_{solvation,\;solid\;hydrate}=-\Delta G_{vaporizaton,water}+\Delta G_{solution}\;\;$$

where

$\Delta G_{solvation,solid\;hydrate}$: Gibbs free energy of solvation of a solute during the formation of solid hydrate;

$\Delta G_{vaporizaton,water}$: Gibbs free energy of vaporization of the solute (water);

$\Delta G_{solution}$: Gibbs free energy of solution;

Thus, for the desolvation of solid hydrate, Equation (2) is not meaningful, while it seems closer to reality to use the following expression:

$$\Delta G_{desolvation}=-\Delta G_{solvation,solid\;hydrate}\;\overset{3}{\Rightarrow }$$

(4) $$\Delta G_{desolvation}=\Delta G_{vaporizaton,water}-\Delta G_{solution}$$

If one wants to apply the criterion for testing the desolvation inability during freeze-drying, then the Gibbs free energy of vaporization should be replaced by the Gibbs free energy of sublimation.

### 3.3. Application of the Criterion to Hydrates of NaCl, MgCl_2_ and CaCl_2_

In order to apply the criterion of Equation (1), the values for the Gibbs free energies of desolvation and decomposition must be calculated. The free energy of desolvation can be calculated from Equation (4) if the free energies of water vaporization and solution are known. The $\Delta G_{vaporizaton,water}$ is available from various sources (e.g., the NIST chemistry webbook) [15]. For the calculation of the $\Delta G_{solution}$, the following reactions were considered:

(R1) $$NaCl_{\left(s\right)}\Leftrightarrow Na_{\left(aq\right)}^{+}+Cl_{\left(aq\right)}^{-}$$

(R2) $$CaCl_{2\left(s\right)}\Leftrightarrow Ca_{\left(aq\right)}^{+2}+2Cl_{\left(aq\right)}^{-}$$

(R3) $$MgCl_{2\left(s\right)}\Leftrightarrow Mg_{\left(aq\right)}^{+2}+2Cl_{\left(aq\right)}^{-}$$

For the calculation of the $\Delta G_{decomposition}$, the following two decomposition reactions were considered for each one of the three “solvents” (NaCl, MgCl_2_, and CaCl_2_).

For NaCl:

(R4) $$2NaCl_{\left(s\right)}\Leftrightarrow 2Na_{\left(s\right)}+Cl_{2\left(g\right)}$$

(R5) $$2NaCl_{\left(s\right)}+H_{2}O_{\left(l\right)}\Leftrightarrow Na_{2}O_{\left(s\right)}+2HCl_{\left(g\right)}$$

For CaCl_2_:

(R6) $$CaCl_{2\left(s\right)}\Leftrightarrow Ca_{\left(s\right)}+Cl_{2\left(g\right)}$$

(R7) $$CaCl_{2\left(s\right)}+H_{2}O_{\left(l\right)}\Leftrightarrow CaO_{\left(s\right)}+2HCl_{\left(g\right)}$$

For MgCl_2_:

(R8) $$MgCl_{2\left(s\right)}\Leftrightarrow Mg_{\left(s\right)}+Cl_{2\left(g\right)}$$

(R9) $$MgCl_{2\left(s\right)}+H_{2}O_{\left(l\right)}\Leftrightarrow MgO_{\left(s\right)}+2HCl_{\left(g\right)}$$

The Gibbs free energies of these reactions (decomposition and solution) were calculated over a temperature range (and pressure of 1 Bar) from the standard (1 Bar and 298.15 K) enthalpies and entropies of reaction. For the sake of simplicity (and as explained below), the standard enthalpies and entropies of the reactions were considered to be constant, thus the Gibbs free energies of the reactions were calculated through the following equation:

(5) $$\Delta G_{reaction}\left(T\right)=\Delta H_{reaction}^{o}-T\times \Delta S_{reaction}^{o}$$

where

$\Delta G_{reaction}\left(T\right)$: The Gibbs free energy of the reaction (decomposition or solution) at some temperature T;

$\Delta H_{reaction}^{o}$: The standard enthalpy of the reaction (decomposition or solution);

$\Delta S_{reaction}^{o}$: The standard entropy of the reaction (decomposition or solution).

The values of the standard enthalpies and entropies of the reactions were calculated, respectively, from the standard enthalpies of formation and the standard molar entropies, which were obtained from the Handbook of Chemistry and Physics [12]. Equation (5) was also used to calculated the Gibbs free energy of the vaporization of water at 1 Bar and various temperatures, by using the values of enthalpy and entropy of the vaporization of water at 1 Bar [15].

Before proceeding, it should be stressed that as for the case of energy of solvation, a similar discussion could be made for the energy of solution. Of course, the energy of solution is not altered if the roles of solvent and solute are reversed, but it surely depends on the composition of the mixture. The approach which was adopted here for the estimation of the free energy of solution, was based on the heats of formation of aqueous ions at concentration of 1 M (not at infinite dilution). Neither the values of 1 M nor any values at infinite dilution are representative for the case of solid hydrate and thus are not truly appropriate to be used in Equation (4). More accurate values could be derived by various modeling approaches based on statistical thermodynamics, quantum mechanics, etc. Such accurate (and time consuming) calculations do not fall within the scope of this work. However, the values for 1 M concentration provide some insights for the order of magnitude of the free energy of solution and since this is used for all substances, it provides comparative information about how strong the solute–water interaction is in each case. In other words, the presented values are approximate values; however, they are useful for extracting valuable conclusions as it will be presented in the following sections by comparing the orders of magnitude of free energy of desolvation and decomposition. Due to the approximate nature of these values, it was not deemed essential to take into account the effect of the specific heat capacities on the enthalpy of the reactions.

Finally, from the literature it is known that the decomposition of the hydrate form of MgCl_2_ is not a single-step reaction, but MgOHCl is formed as an intermediate product and is stable over some temperature range. Unfortunately, as in the case of various organic substances, no data could be found for the MgOHCl. However, the study of the overall reaction instead of the two intermediates is of value since the Gibbs free energy of the overall reaction is the sum of the intermediate reactions. Thus, by studying the overall reaction, conclusions can be derived; simply, it cannot be argued which of the intermediate reaction is the governing stage of the overall reaction.

## 4. Results and Discussion

### 4.1. Theoretical Aspects of Desolvation Ability/Inability at Atmospheric Pressure

In Table 1, the standard Gibbs free energies of decomposition, solution, and water vaporization are presented. Initially, we will focus our discussion on the case of NaCl. Regarding the two possible decomposition reactions, it can be seen that both of them exhibit positive values for the standard Gibbs free energies. However, the decomposition reaction that involves water as a reactant (reaction R5) exhibits a much lower value than the other reaction (reaction R4), thus, this reaction is the preferred one and its value (+439 kJ/mol) will be used in Equation (1) for the case of NaCl.

The Gibbs free energy of desolvation for the case of NaCl, according to Equation (4), is equal to 8 − (−9) = +17 kJ/mol. By substituting the respective values in Equation (1), it follows that the value of DI for NaCl (at 1 Bar and 298.15 K) is equal to +17 − 439 = −422 kJ/mol. Thus, the value of DI for NaCl is highly negative indicating a very low probability for the exhibition of dehydration inability and on the contrary it points out that it should be possible to dehydrate NaCl. In the literature, the Gibbs free energy of hydration of NaCl has been reported to be around −750 kJ/mol [1]. If we use the opposite of this value in Equation (1), instead of using the Gibbs free energy of desolvation as proposed in this study (Equation (4)), the value of DI is equal to: +750 − 439 = +311 kJ/mol. This would mean that it is easier to decompose NaCl than to dehydrate it. However, such a conclusion is contradictory to experimental observations and it is widely accepted that the solid dehydrated form of NaCl exists [16]. The above point out that the proposed approach for the calculation of the Gibbs free energy of desolvation is more proper to be used for the implementation of the criterion (Equation (1)).

**Figure 1:**
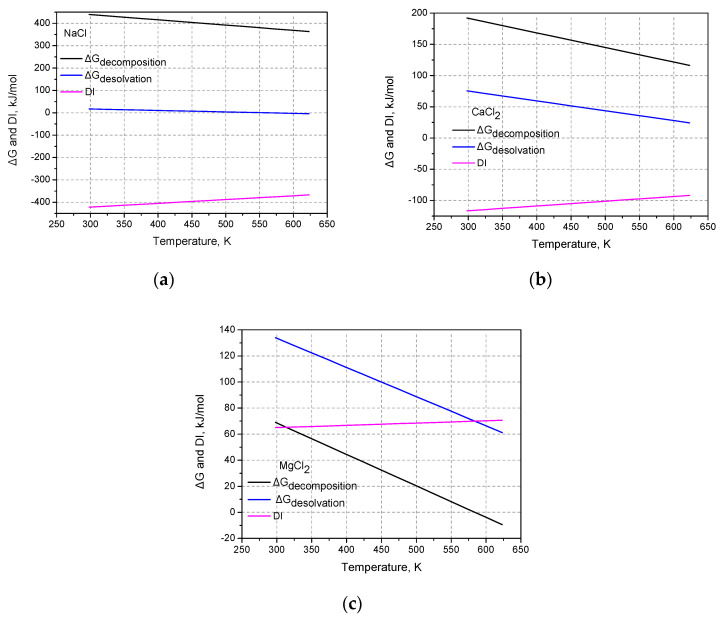
Gibbs free energies of decomposition, desolvation and values of DI in the temperature range 250–650 K: (**a**) NaCl, (**b**) CaCl_2,_ and (**c**) MgCl_2_.

As for the case of NaCl, also for the cases of CaCl_2_ and MgCl_2_, the decomposition reactions at 1 Bar and 298.15 K are characterized by positive Gibbs free energies values, but the least positive value regards the reactions in which water is involved. Thus, the Gibbs free energy values of these reactions were taken into account in Equation (4). The value of DI (at 1 Bar and 298.15 K) for CaCl_2_ is [+8 − (−67)] −192 = −117 kJ/mol while the respective value for MgCl_2_ is [+8 − (−125)] − 69 = +64 kJ/mol. Based on these values, it can be concluded that the thermal dehydration of CaCl_2_ hydrate should be possible in contrast to the case of MgCl_2_ hydrate. These conclusions are in agreement with the experimental observations for CaCl_2_ [4,17] and MgCl_2_ [5,8]. By using Equation (5), we can have an estimation for the value of DI at 1 Bar and various temperatures. These values along with the corresponding values of $\Delta G_{decomposition}$ and $\Delta G_{desolvation}$ for NaCl, CaCl_2,_ and MgCl_2_, are presented, respectively, in Figure 1a, Figure 1b and Figure 1c.

As can be seen in Figure 1, in all three cases, the DI increases mildly with temperature. This mild increase, however, does not alter the conclusion which is derived from the standard values at 298.15 K. Both the Gibbs free energies of decomposition and desolvation decrease with the increase in temperature. From Figure 1, it is clear that in the case of MgCl_2_, not only the Gibbs free energies of decomposition, desolvation are of the same order of magnitude but the one of desolvation is higher than the one of decomposition. On the contrary, for the cases of NaCl and CaCl_2_, the free energy of decomposition is quite higher than the one of desolvation. As a consequence, the DI values for NaCl and CaCl_2_ are highly negative, which indicate that for these two substances the desolvation is favored over decomposition. On the contrary, the positive DI value for the case of MgCl_2_ points out that for this substance the decomposition is favored over desolvation. These conclusions are in agreement with various experimental results (see Section 1).

Some of the Gibbs free energies become negative upon heating (the $\Delta G_{desolvation}$ for NaCl, and the $\Delta G_{decomposition}$ for MgCl_2_). Of course, more accurate values could be calculated, e.g., by considering the temperature variation of the specific heat capacities. It is worth mentioning that even in the case in which the Gibb free energy is positive (that is, the reaction does not proceed spontaneously), this does not mean that the yield of the reaction is zero, e.g., in reversible reactions such as esterification. If both of the reactions (decomposition and desolvation) are characterized by positive Gibbs free energy values, then, the one with the lowest value is more likely to occur since it is less unfavored.

### 4.2. Further Discussion Regarding the Desolvation Inability and Freeze-Drying

Despite the assumptions and simplifications that were adopted in the analysis of the previous section (e.g., the neglect of the effect of specific heat capacity on the enthalpy of reaction or the incorrect value for the energy of solution), valuable conclusions can be derived. Based on Equation (1), the exhibition of desolvation inability is favored by high values of energy of desolvation and low values of energy of decomposition. The Gibbs free energy of desolvation is pretty much of the same order of magnitude in all three cases while the Gibbs free energy of decomposition of MgCl_2_ is countably lower compared to the ones of all other decomposition reactions (including the reaction of MgCl_2_ into Cl_2_ and Mg). The interaction of such inorganic salts with water (ion–dipole interaction) is perhaps the strongest physical interaction and stronger than hydrogen bonding. Thus, the order of magnitude of desolvation energy cannot be much higher than the values presented here (even if we take into account the specific heat capacities, etc.). In other words, the low value of the Gibbs free energy of decomposition seems to be the governing factor for the exhibition of solvation inability.

Temperature surely affects the values of the Gibbs energies but the order of magnitude of these values is the same over a wide range of temperatures. The same could be claimed about the effect of pressure. Thus, if the free energy of decomposition is quite lower than the one of desolvation at standard state conditions, then it would be expected that this would be the case also for a range of pressures and temperatures. In other words, at low pressure and temperature (which are used in freeze-drying) the decomposition may still be favored over desolvation. Of course, the low temperatures that are used in freeze-drying will have a tremendous effect on the kinetics of the decomposition reaction, and it is widely known that the reaction rate decreases exponentially with the decrease in temperature. However, during heating, the decomposition of a hydrate occurs within some seconds/minutes, while freeze-drying lasts for many hours. Thus, the reaction rate during freeze-drying should be orders of magnitude lower than the one during heating, but the available time in order for decomposition to occur is orders of magnitude higher in the case of freeze-drying. Thus, it would not be surprising if some traceable decomposition occurs during 20–30 h of freeze-drying.

In addition, as mentioned in the Introduction in various cases (gallic acid and cellulose esters) the primary decomposition reaction is an esterification reaction. It was pointed out the easiness of the occurrence of such reaction must be related to the low value of the enthalpy of esterification, which was estimated to be comparable to the enthalpy of a hydrogen bond (~15 kJ/mol) [9]. If instead of OH, an amine group reacts with a COOH group then a peptide bond is formed, and a water molecule is produced as in the esterification reaction. Both of these reactions are reversible. Similarly to the esterification reaction, the condensation reaction is also characterized by a low value of enthalpy of reaction, which was calculated by using the average bond energies [18]. In addition, it was shown that the low value of enthalpy of the condensation reaction is responsible for qualitative shifts, e.g., the condensation reaction may shift from slightly endothermic to slightly exothermic under small variations (20–50 °C) in temperature [18]. Also, the peptide (and other chemical) bonds do not vibrate with the exact same frequency, and this is translated to different bond energy. Thus, the same reaction, for some bonds may be endothermic and for other bonds it may be exothermic. Again, the variation of the stretching vibration frequency (that is the variation of the bond strength) can cause such a qualitative shift due to the low value of the enthalpy of the reaction [18]. According to the Le Chatelier’s principle, the above means that not all the bonds will exhibit the same behavior upon exposure to various temperatures. For example, during freeze-drying, the exothermic reaction will be favored while upon heating the endothermic reaction will be favored. In addition, in a solid hydrate, if water removal is initiated (either by heating or by freeze-drying) this shifts the reaction towards the esterification (or condensation) direction as new water is produced in order to balance the removal of the originally present water.

Based on the above arguments, it is predicted that decomposition during freeze-drying is possible, especially for substances like hydroxy-acids and amino-acids where reactions of very low enthalpy are involved, e.g., esterification. In what follows, the experimental results for the freeze-drying of MgCl_2_·6H_2_O and gallic acid are presented and discussed.

### 4.3. Experimental Aspects Regarding Desolvation Inability and Freeze-Drying

XRD (as FTIR) is a volume-dependent technique which is used for quantitative analysis. The intensity of the XRD peak depends on the volume fraction of the spacing which is responsible for the diffraction peak. The subtraction of two graphs is common approach that is used in FTIR and facilitates the recognition of the spectrum differences between two samples. In the subtracted spectrum a negative peak indicates decrease of the group (or spacing) responsible for the peak while correspondingly a positive peak indicates an increase in the content. Thus, by the existence of positive and negative peaks in the subtracted spectrum of two samples, their differences can be readily recognized. Here we used the approach of graph subtraction for both XRD and FTIR in order to detect differences in the chemical structure of raw and freeze-dried samples. In what follows, we present and discuss these results.

#### 4.3.1. Freeze-Drying of MgCl_2_·6H_2_O

**Figure 2:**
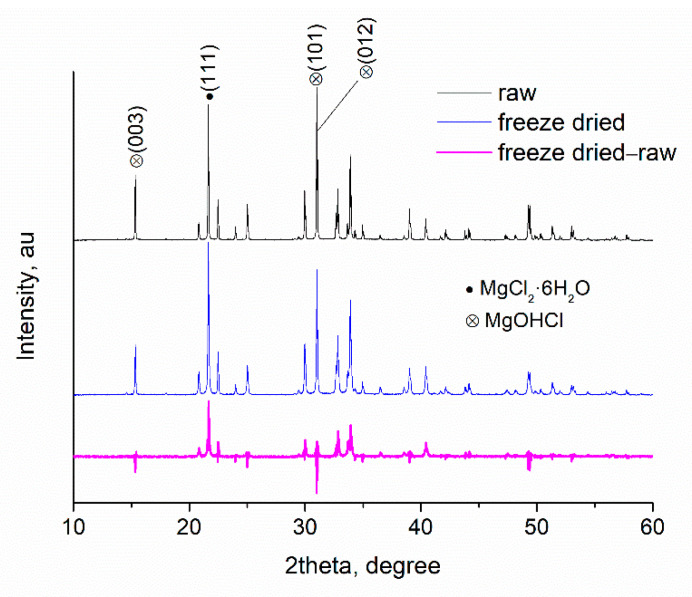
XRD graphs of raw and freeze-dried MgCl_2_·6H_2_O and their subtracted graph.

In Figure 2, the XRD graphs of MgCl_2_·6H_2_O before and after freeze-drying are presented. The graphs are pretty similar and only some minor alterations in the intensity of some peaks can be observed. To facilitate the evaluation of these alterations, the graphs have been subtracted (graph of the freeze-dried sample minus the graph of the raw sample) and the subtracted graph is also presented in Figure 2. Most of the peaks are related to MgCl_2_·6H_2_O and in Figure 2, suggestively, the main peak (with the highest intensity at 21.64° corresponding to the (111) plane [19]) is highlighted. The peak at 15.35°, however, is not a diffraction peak of the crystal lattice of MgCl_2_·6H_2_O. This peak is the peak with the highest intensity of MgOHCl [19] corresponding to the (003) plane (also highlighted in Figure 2). The peak of MgOHCl with the second highest intensity is a double peak at around 31° corresponding to the (101) and (012) planes [19]. These peaks overlap with the peaks of MgCl_2_·6H_2_O (highlighted in Figure 2). The main peak of MgOHCl is clearly visible in the graph of the freeze-dried sample. In the subtracted graph this peak is negative, and a double negative peak also exists at 31°. In the same region, as well as at 21.64°, and at other regions, positive peaks exist (related to the main phase of MgCl_2_·6H_2_O). These clearly show that after freeze-drying the content of MgOHCl has decreased and the content of MgCl_2_·6H_2_O has increased. Of course, the increase of MgCl_2_·6H_2_O may be relative (not actual) due to the decrease of MgOHCl and not due to actual formation of new MgCl_2_·6H_2_O.

The slight decrease of the MgOHCl could be considered as weak evidence that this substance decomposed to some low extent during freeze-drying. There are two possibilities: (a) MgOHCl decomposed to MgO and HCl and (b) MgOHCl decomposed to some hydrate form of MgCl_2_. In the XRD graphs, there are no signs to support the presence of MgO (e.g., its main diffraction peak is located at 42.9° corresponding to the (200) plane [20]) and no peak can be detected at this angle. Perhaps any traces of MgO could be below the detection limit of the instrument. Since the peaks of MgCl_2_ have increased, the second scenario seems more probable, and it should be stressed that this cannot be considered as a “traditional” (thermophysical) transformation between polymorphs. It is recalled that MgOHCl is an intermediate product of MgCl_2_ hydrolysis. In other words, the sample under study seems to be a complex hydrate of MgCl_2_ and MgOHCl (either it was produced as such, or some hydrolysis took place during storage at room temperature). The relative amount of the two substances in this complex hydrate has changed by freeze-drying and no evidence for full dehydration can be found.

#### 4.3.2. Freeze-Drying of Gallic Acid

In a previous work [11], the thermal behavior of gallic acid was thoroughly studied by using raw gallic sample and a sample that was recrystallized from D_2_O. This allowed for the assignment of various IR bands. It was reported that the mass loss of gallic acid around 85 °C is not due to simple water vaporization (removal of hydrated water) but due to the occurrence of decomposition of gallic acid, and actually the water content is increased by heating, since the primary decomposition reaction was found to be the esterification reaction of the OH and COOH groups of gallic acid [11]. A similar increase in the water content by heating, due to an esterification reaction, has also been reported to occur in cellulose esters [9]. It should be stressed that the gallic acid used in that work (as well as in the current work) is not “officially” a hydrate form of gallic acid. However, the existence of water was proven [11], by the presence of the bands at 3495 and 3280 cm^−1^ assigned to the O–H stretching vibrations and the bands at around 1650 cm^−1^ assigned to H–O–H bending [21].

**Figure 3:**
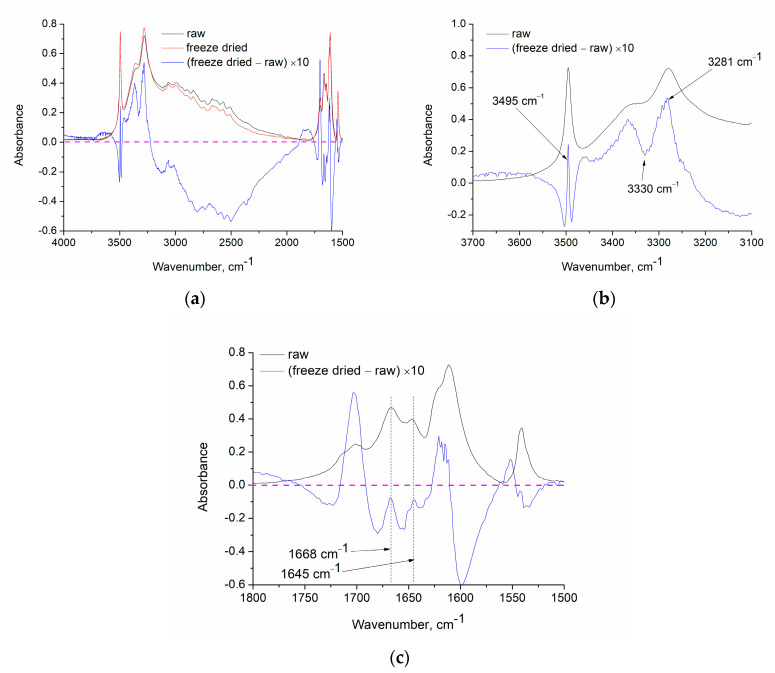
FTIR spectra of raw gallic acid, freeze-dried gallic acid and their subtracted spectrum multiplied by 10: (**a**) all three spectra in the region 1500–4000 cm^−1^, (**b**) FTIR spectra of raw gallic acid and subtracted spectrum in the region 3100–3700 cm^−1^, and (**c**) FTIR spectra of raw gallic acid and subtracted spectrum in the region 1500–1800 cm^−1^.

In Figure 3a, the FTIR spectrum of raw gallic acid, freeze-dried gallic acid and their subtracted spectrum (the spectrum of the freeze-dried sample minus the spectrum of the raw sample multiplied by 10) are presented. In the spectrum of the raw sample, the sharp peak at 3495 cm^−1^ is clearly visible while the peak at 3280 cm^−1^ is overlapped by carboxylic O-H vibrations [11]. Around 1650 cm^−1^, there are two bands located at 1668 and 1645 cm^−1^. The ratio of the areas of these bands was found to be practically equal to 3/1, which is the ratio of the phenolic OH to carboxylic OH in gallic acid. This was interpreted as follows: Since there are three phenolic OH groups in gallic acid and on COOH group, it is three times more probable for water molecules to form a hydrogen bond with phenolic OH. Thus, the bands at 1668 and 1645 cm^−1^ were assigned to the H–O–H vibration of water hydrogen bonded, respectively, to phenolic OH and COOH [11]. These bands are clearly visible also in the spectrum of the freeze-dried sample (Figure 3a). Thus, there is no evidence that could support that water was removed from gallic acid after 21 h of freeze-drying.

In general, the spectra of the raw and the freeze-dried sample are pretty similar. However, in their subtracted spectrum, various relative changes can be realized. A negative peak around 3500 cm^−1^ is visible. This peak could be assigned to phenolic O–H stretching since it is known that such O–H vibrations appear at these high wavenumbers while O–H of COOH generally appear at lower wavenumbers [21]. This decrease in the phenolic OH, could be understood if decomposition (e.g., esterification reaction) took place during freeze-drying. In addition, as can be seen in Figure 3b, within this negative peak, the signal becomes positive at 3495 cm^−1^. This points out that the (relative) content of water has increased or that it has not decreased as much as the other groups, e.g., some of the originally present water was removed and new water was formed. Of course, in such a case, the other peaks which are related to water should also be positive. Indeed, at 3280 cm^−1^ (Figure 3b) and at 1668 and 1645 cm^−1^ (Figure 3c) the signal is positive. These further support the occurrence of esterification. Also, a reduction of COOH groups should occur if esterification takes place. Indeed, around 3330 and 3150 cm^−1^ negative peaks can be observed (Figure 3b). These O–H vibrations are related to groups which are strongly (hydrogen) bonded and are known to appear at lower wavenumbers than the free ones [21]. The positive peak at 3350 cm^−1^ could be attributed to an increase in free O–H (COOH) groups. The reduction of COOH groups can also be supported by the C=O reduction (Figure 3c). More precisely, if esterification occurs, then, besides the C=O vibration of the COOH group, an additional C=O vibration of the produced ester should appear. Indeed, at 1713 cm^−1^, a negative peak can be observed (Figure 3c), while a positive peak at lower wavenumber (1700 cm^−1^) has appeared. Similar shifts (but at an even lower wavenumber <1700 cm^−1^) were observed in the gallic acid sample heated at 100 °C (either the raw sample or the recrystallized one from D_2_O) [11]. Finally, in the region 1500–1650 cm^−1^, various vibrations related to the benzene ring occur, e.g., C=C vibrations [21]. If a dimer of gallic acid is formed through esterification, then it would be expected that the benzene ring vibrations would be different. Indeed, such vibrations have shifted to higher wavenumbers in the freeze-dried sample, as can be concluded by the negative and positive peaks in this region (Figure 3c).

As a summary, minor but traceable alterations in the chemical structure of gallic acid after freeze-drying can be claimed. Also, it cannot be supported that full water removal has occurred. The alterations in the structure are not as severe as the ones invoked by heating at 100 °C [11]. There are at least two factors potentially contributing to this. First, there are three OH groups in gallic acid (3,4,5-trihydroxy benzoic acid) at positions 3, 4, and 5. At different temperatures (e.g., at 100 and −5 °C), perhaps a different OH is involved in the esterification reaction. Second, the reaction rate at sub-zero temperature is much lower leading to a lower reaction yield. In the case of heating, the oligomers of gallic acid may be of higher molecular weight, e.g., some heptamers are produced, while in the case of freeze-drying, e.g., dimers are formed. Thus, in the case of freeze-drying, the alterations in the chemical structure are not such severe and are not exactly the same as the ones during heating.

## 5. Conclusions

From the available literature data, it is clear that, similarly to the melting inability of solids, a desolvation inability is exhibited by some solids, and no full dehydration can occur prior to decomposition; that is, either not all the solvent will be removed, or decomposition will occur. Although here we focused on hydrates, there is no reason to assume that this behavior will not be exhibited by other solvates. Indeed, the solvation of cellulose acetate by acetic acid can be considered as such an example and is responsible for the reported thermal behavior of cellulose acetate. An explanation for the behavior of desolvation inability was provided based on the comparison of the Gibbs free energies of decomposition and desolvation, and a corresponding criterion/index was proposed in order to predict this behavior. A new approach for the expression of the Gibbs free energy of desolvation was proposed by reversing the roles of the solute and solvent and by regarding water as the solute, otherwise, very high values of Gibbs free energy of desolvation are obtained and misleading conclusions may be reached. Briefly, in certain cases, the Gibbs free energy of decomposition is lower than the Gibbs free energy of desolvation and thus decomposition is more favored (or less unfavored) compared to dehydration. By this simple concept, the thermal behavior of three inorganic hydrates of NaCl, CaCl_2,_ and MgCl_2_ can be understood and explained. The differences in the Gibbs free energies of decomposition and desolvation, are countable (in some cases they differ in order of magnitude), and in such cases a prediction for the dehydration ability/inability can be derived by the standard values at 298.15 K. If the Gibbs free energy of decomposition is low, this favors the desolvation inability. The low enthalpy of the esterification reaction also seems to contribute to the desolvation inability of some organic compounds, e.g., gallic acid. Decomposition instead of desolvation can occur even at sub-zero temperatures during freeze-drying, as shown in this study for gallic acid.

## Figures and Tables

**Table 1:** Standard (at 1 Bar and 298.15 K) Gibbs free energies of decomposition, solution, and water vaporization.

Reaction	Type	$\Delta G^{o}$, kJ/mol
$2NaCl_{\left(s\right)}\Leftrightarrow 2Na_{\left(s\right)}+Cl_{2\left(g\right)}$	decomposition	+768
$2NaCl_{\left(s\right)}+H_{2}O_{\left(l\right)}\Leftrightarrow Na_{2}O_{\left(s\right)}+2HCl_{\left(g\right)}$	decomposition	+439
$NaCl_{\left(s\right)}\Leftrightarrow Na_{\left(aq\right)}^{+}+Cl_{\left(aq\right)}^{-}$	solution	−9
$CaCl_{2\left(s\right)}\Leftrightarrow Ca_{\left(s\right)}+Cl_{2\left(g\right)}$	decomposition	+749
$CaCl_{2\left(s\right)}+H_{2}O_{\left(l\right)}\Leftrightarrow CaO_{\left(s\right)}+2HCl_{\left(g\right)}$	decomposition	+192
$CaCl_{2\left(s\right)}\Leftrightarrow Ca_{\left(aq\right)}^{+2}+2Cl_{\left(aq\right)}^{-}$	solution	−67
$MgCl_{2\left(s\right)}\Leftrightarrow Mg_{\left(s\right)}+Cl_{2\left(g\right)}$	decomposition	+592
$MgCl_{2\left(s\right)}+H_{2}O_{\left(l\right)}\Leftrightarrow MgO_{\left(s\right)}+2HCl_{\left(g\right)}$	decomposition	+69
$MgCl_{2\left(s\right)}\Leftrightarrow Mg_{\left(aq\right)}^{+2}+2Cl_{\left(aq\right)}^{-}$	solution	−125
$H_{2}O_{\left(l\right)}\Leftrightarrow H_{2}O_{\left(g\right)}$	vaporization	+8

## Data Availability

Data are contained within the article.
